# Supplementary material for: Electron transport and visible light absorption in a plasmonic photocatalyst based on strontium niobate
Source: Nat Commun. 2017 Apr 19;8:15070. doi: 10.1038/ncomms15070 (PMC5399282; doi:10.1038/ncomms15070)
Supplement: Supplementary Information — Supplementary Figures, Supplementary Tables, Supplementary Notes and Supplementary References [file ncomms15070-s1.pdf]

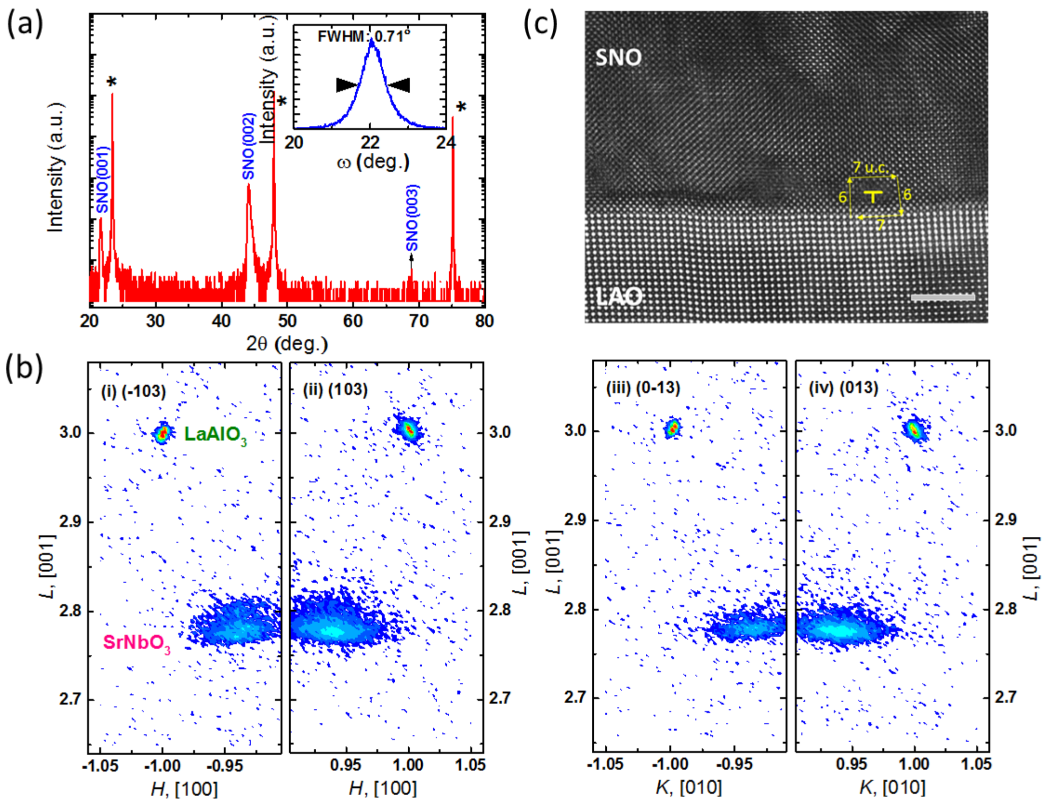

Supplementary Figure 1 | XRD patterns and TEM image of the $SrNbO_3$ film grown on $LaAlO_3$(001) substrate. The film was deposited under oxygen partial pressure of 5×10^{-6} Torr. (a) θ-2θ scan, where * indicates the signals of the substrate. The inset shows the rocking curve. (b) RSMs on (-103), (103), (0-13) and (013) reflections from the substrate and the film. (c) High-resolution TEM image of a square region. The scale bar is 4 nm. The shaped open circuit area by yellow arrows highlights an edge dislocation core (type a[100]p).

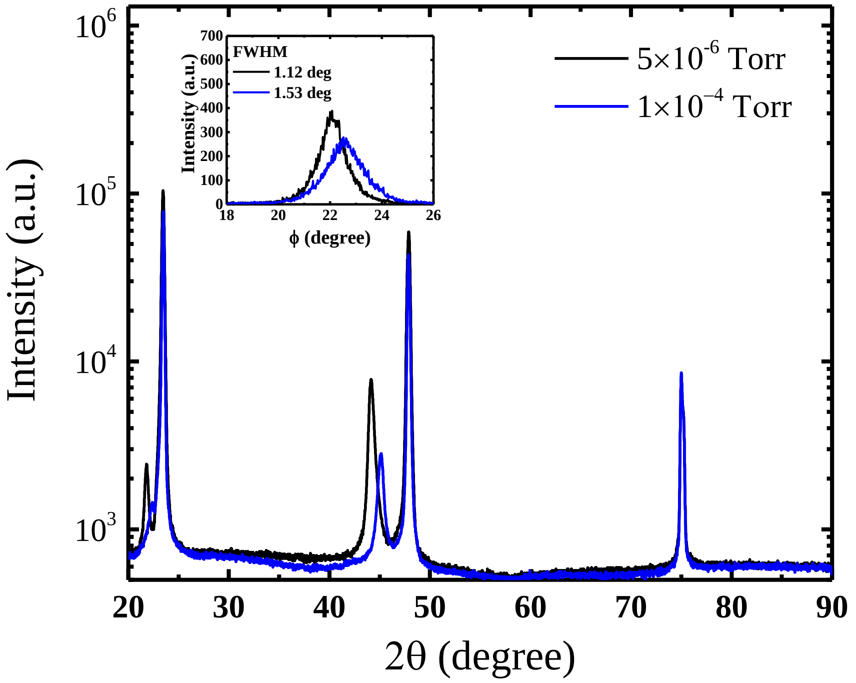

Supplementary Figure 2 | XRD spectrum of $SrNbO_{3+\delta}$ thin films deposited at various oxygen partial pressures.

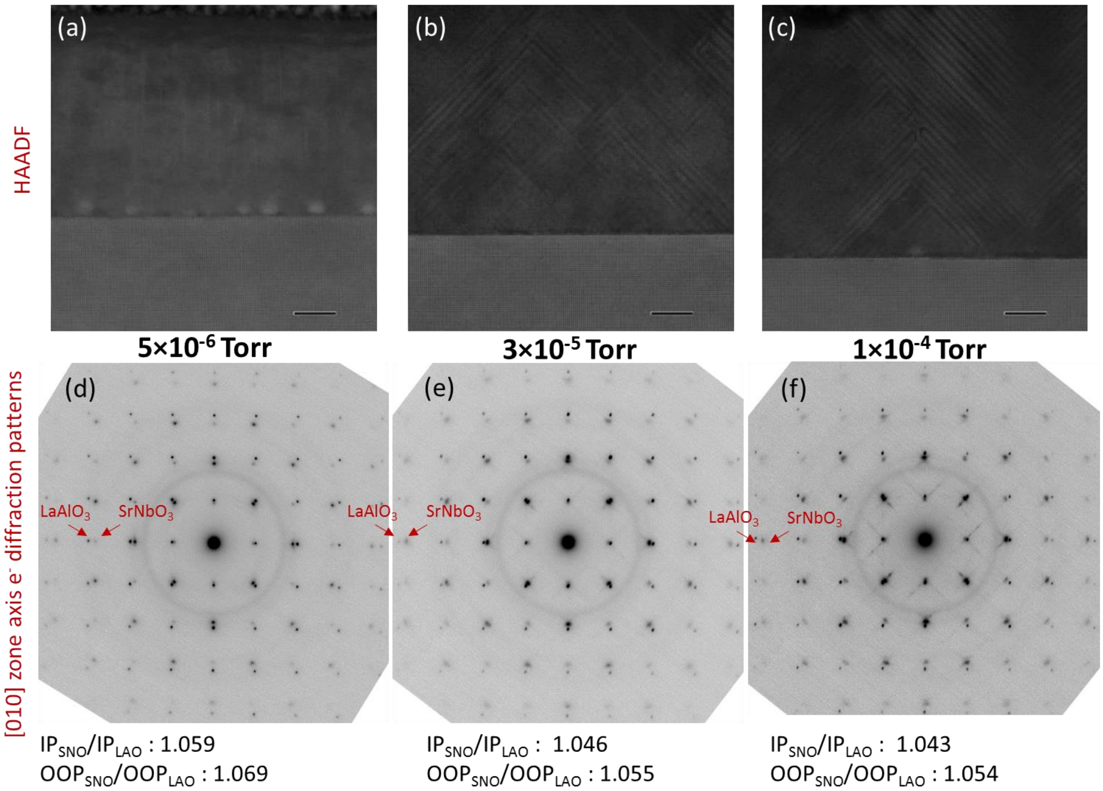

Supplementary Figure 3 | Structure changes of $SrNbO_{3+\delta}$ thin films induced by the oxygen partial pressures when deposited. (a-c) Cross-sectional TEM images $SrNbO_{3+\delta}$ thin films deposited at 5×10^{-6} Torr, 3×10^{-5} Torr and 1×10^{-4} Torr, respectively. The scale bar shown in the right bottom corner is 8 nm. (d-f) Electron diffraction pattern of $SrNbO_{3+\delta}$ thin films deposited at 5×10^{-6} Torr, 3×10^{-5} Torr and 1×10^{-4} Torr, respectively.

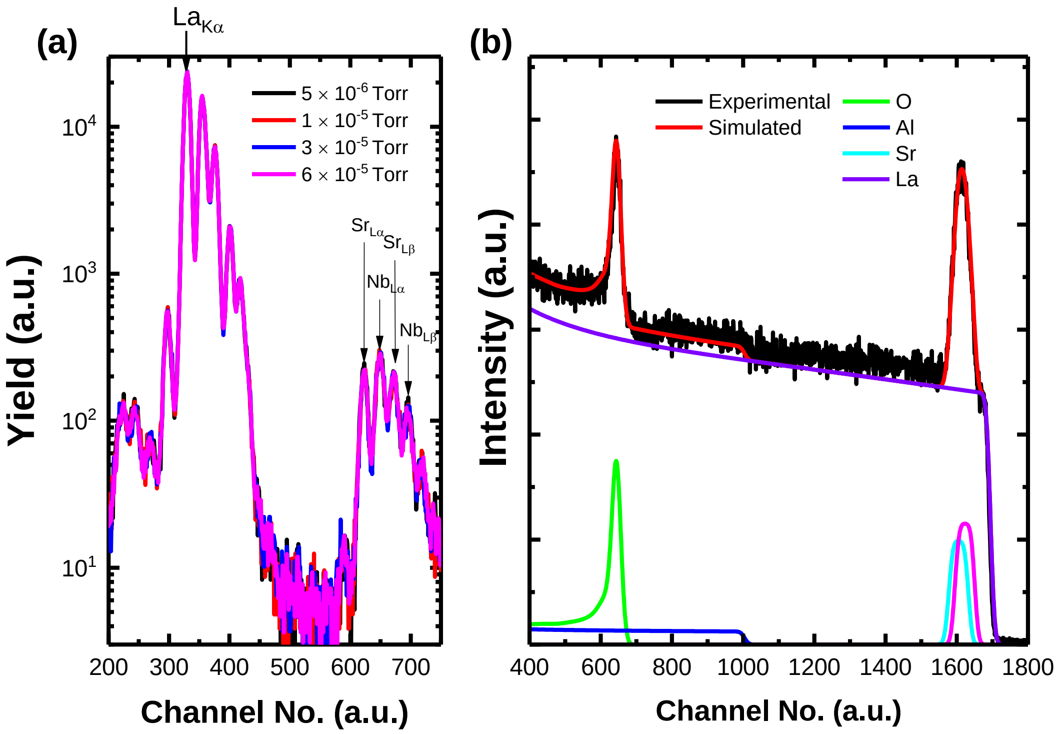

Supplementary Figure 4 | PIXE and RBS spectrums of $SrNbO_3$ films grown on $LaAlO_3$ substrate. (a) 2 MeV PIXE spectrum. (b) 3.04 MeV oxygen resonance enhanced RBS spectrum showing Sr, Nb and Oxygen spectra for the film on LaAlO3 substrate. The film was prepared at 5×10^{-6} Torr and the fitted composition is nominally SrNbO3. The estimated error in the oxygen measurement is $\pm$ 5% and that for Sr to Nb ratio is about $\pm$ 2%.

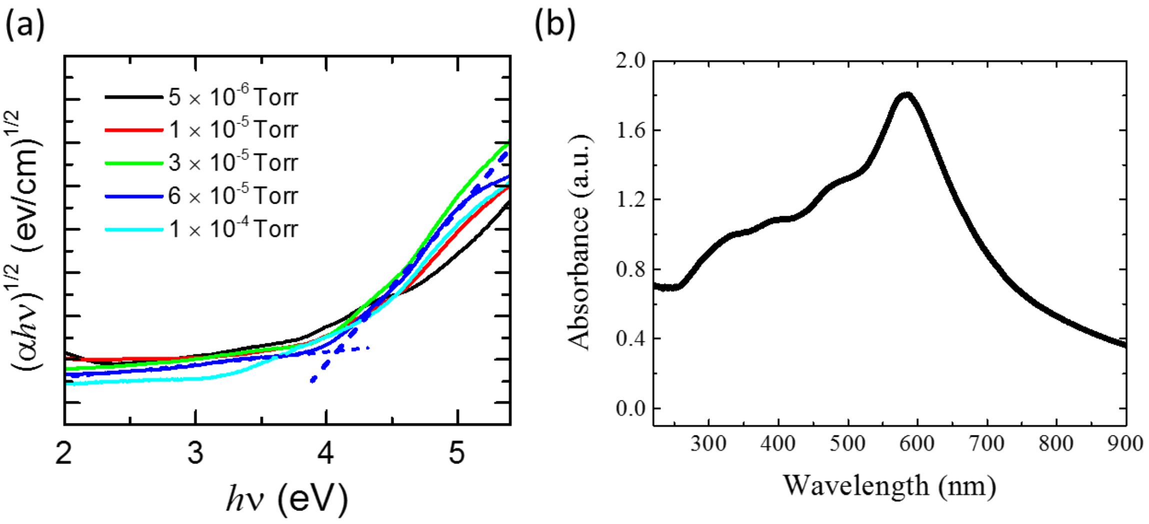

Supplementary Figure 5 | (a) The Tauc-indirect plot of the films deposited at various oxygen partial pressures, an optical band gap of 4.1 eV could be obtained. (b) The absorbance spectrum obtained from the reflectance spectrum of the $SrNbO_3$ film deposited at $5{\times}10^{-6}$ Torr.

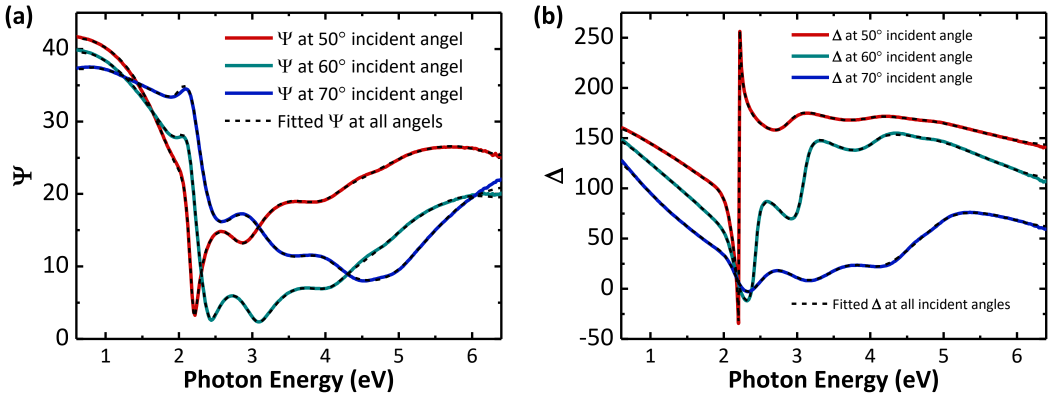

Supplementary Figure 6 | Analysis of spectroscopic ellipsometry data of the 5×10^{-6} Torr sample. a, Analysis of Ψ of the 5×10^{-6} Torr sample using uniaxial anisotropic mode. **b**, Analysis of Δ of the 5×10^{-6} Torr sample using uniaxial anisotropic mode.

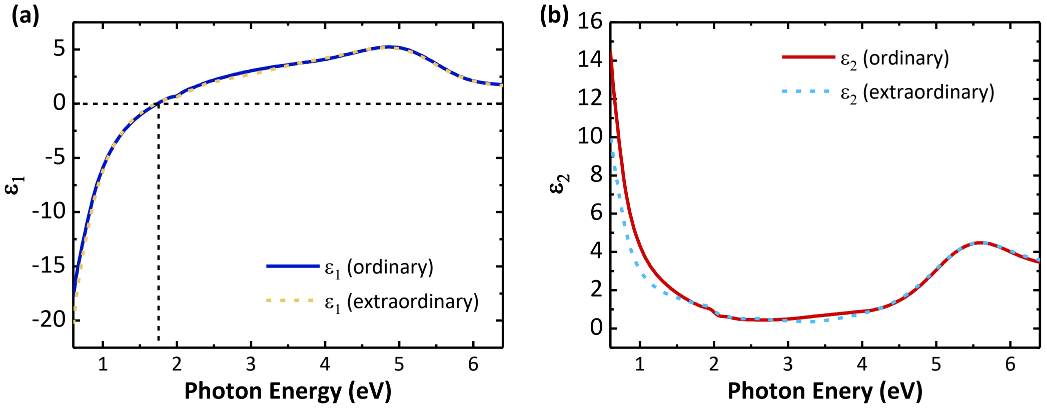

Supplementary Figure 7 | Complex dielectric function of the 5×10^{-6} Torr film. a, Real part of ordinary and extraordinary complex dielectric function, $\varepsilon_1(\omega)$, spectra of the 5×10^{-6} Torr film. Vertical dashed lines indicate the zero-crossings of ordinary and extraordinary $\varepsilon_1(\omega)$ of the film. b, Imaginary part of ordinary and extraordinary complex dielectric function, $\varepsilon_2(\omega)$, spectra of the 5×10^{-6} Torr film.

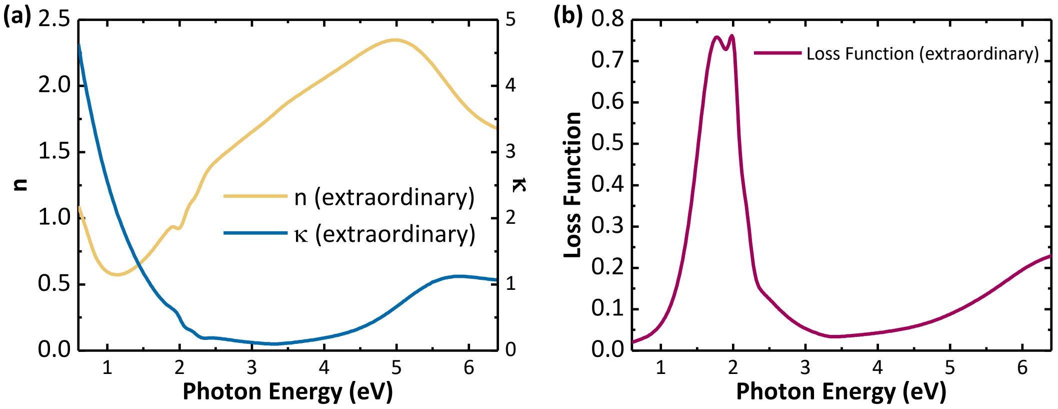

Supplementary Figure 8 | Extraordinary complex refractive index and loss function of the 5×10^{-6} Torr film. a, Frequency-dependent extraordinary complex refractive index, $\tilde{n}(\omega) = n(\omega) + \kappa(\omega)$, of the 5×10^{-6} Torr film. **b**, Extraordinary loss function spectra of the 5×10^{-6} Torr film.

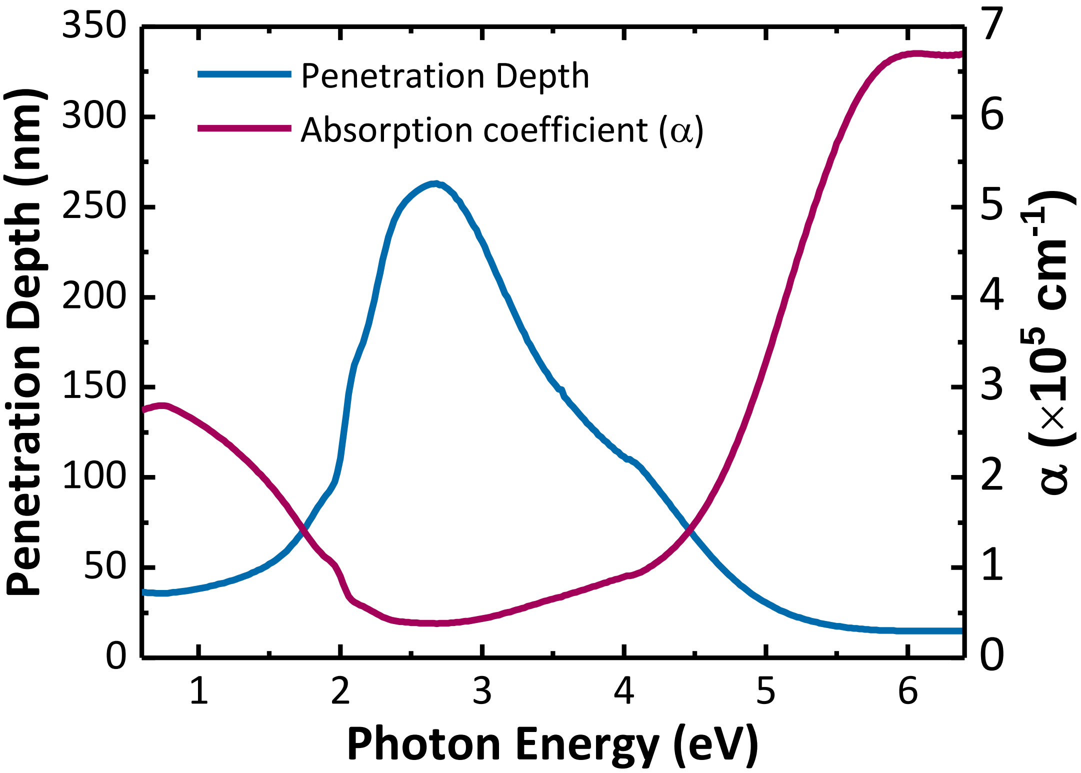

Supplementary Figure 9 | Absorption coefficient, $\alpha(\omega)$, and photon penetration depth of the 5×10^{-6} Torr $SrNbO_3$ film. The vertical black dashed line indicates the bulk plasmon energy of ~1.8 eV, and the horizontal black dashed line indicates the photon penetration depth at the bulk plasmon energy, which is ~80 nm.

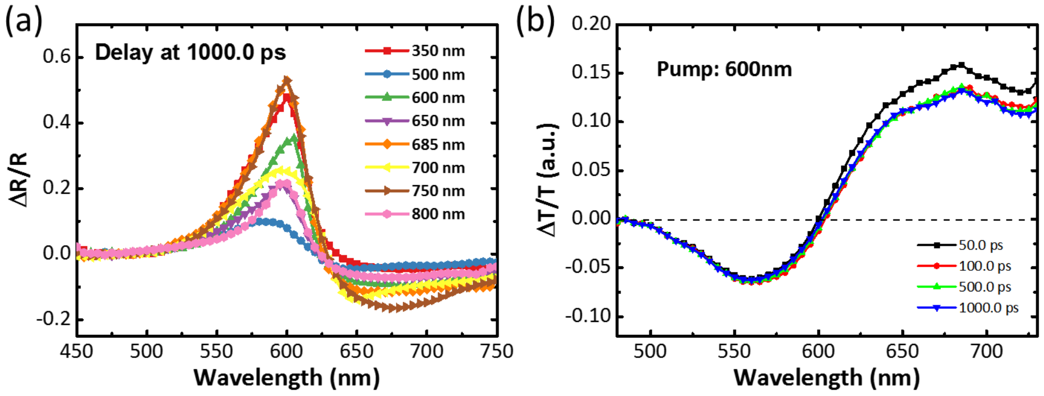

Supplementary Figure 10 | (a) The differential reflection spectra (ΔR/R) of $SrNbO_3$ with various excitation wavelengths and the time delay at 1000.0 ps. (b) The transmittance spectra (ΔT/T) of the $SrNbO_3$ film with various time delays and the pump light at 600 nm.

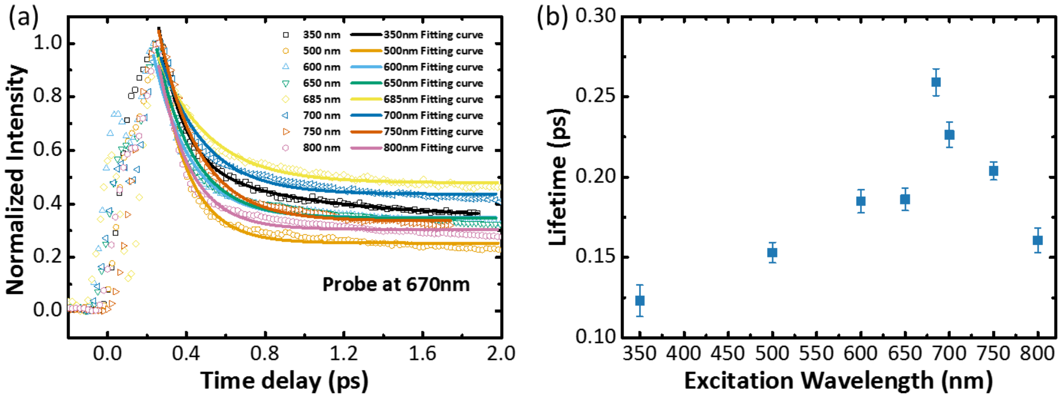

Supplementary Figure 11 | The dynamics of the hot electrons in $SrNbO_3$ within the time delay of 2.0 ps. (a) The time-resolved differential reflection spectra of $SrNbO_3$ with various excitation (pump) wavelengths. (b) The lifetime of the process during the first 2.0 ps of various excitation wavelengths.

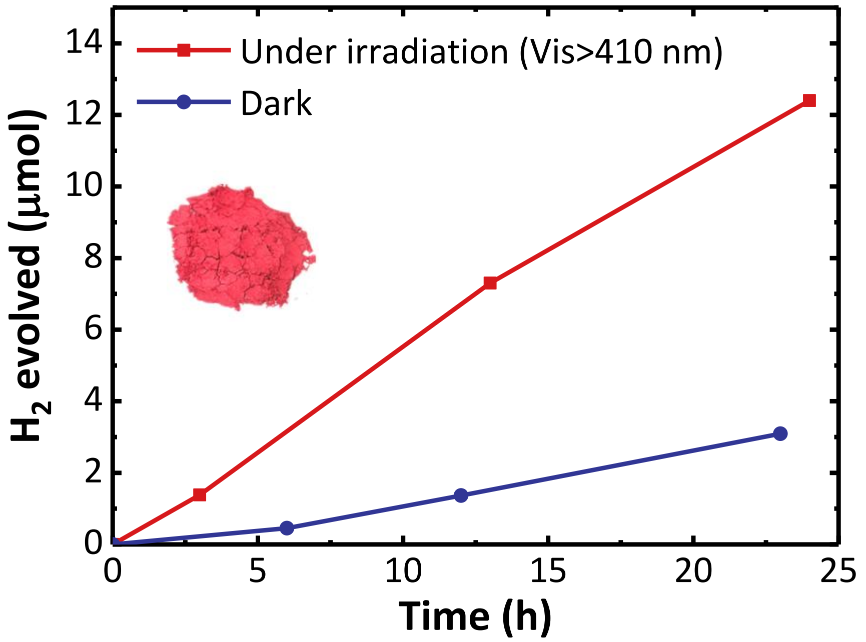

Supplementary Figure 12 | Time courses of H_2 gas evolution of as-prepared 50 mg $SrNbO_3$ powder in aqueous oxalic acid solution (0.025M, 100 ml) under visible light irradiation (red) and without irradiation (blue). 1 wt. % of Pt is used as co-catalyst. The inset shows the photo of the $SrNbO_3$ powders.

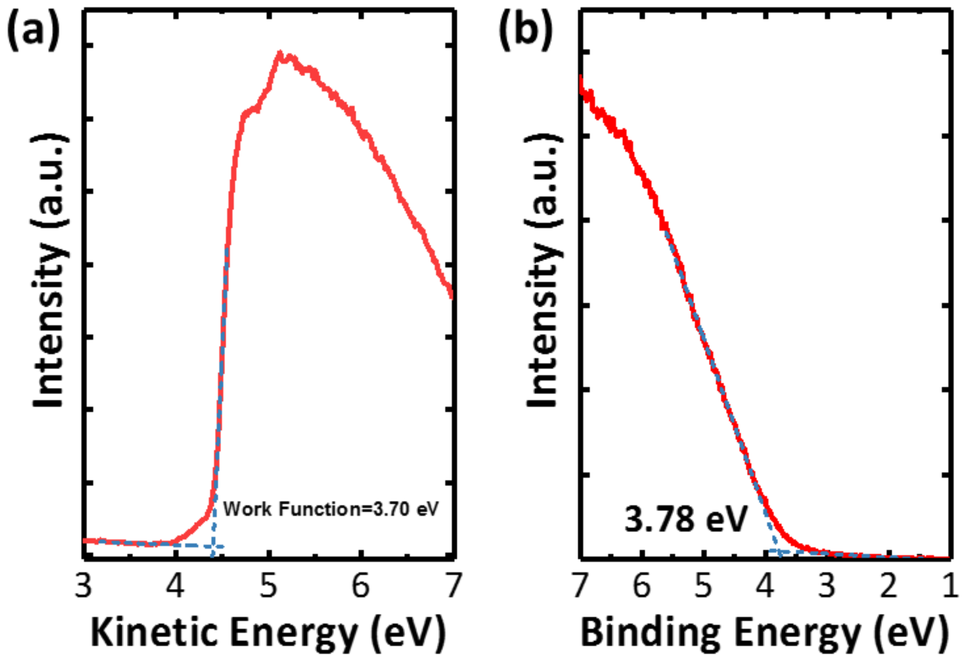

Supplementary Figure 13 | The UPS spectra of $SrNbO_3$ thin film shows that the Fermi level position is at 3.70 eV (a) and the B_{-1} band onset position is 3.78 eV below the Fermi level (b). There is no mid-gap state between B_{-1} band and conduction band.

Supplementary Table 1 | Lattice parameters of $SrNbO_{3+\delta}$ thin films

	10^{-6}	3×10^{-5}	10^{-4}
IP lattice a_{IP}^{*}	4.02Å	3.96Å	3.95Å
OOP lattice a_{OOP}^{*}	4.05Å	4.00Å	3.99Å

*Given $LaAlO_3$ lattice constant of 3.79Å

Supplementary Table 2 | The composition of films deposited at various oxygen pressure

O2 Pressure (Torr)	Composition from RBS		
	**Sr**	**Nb**	**O**
5×10^{-6}	1	1	3
3×10^{-5}	1	1	3.35
1×10^{-4}	1	1	3.55

Supplementary Table 3 | Fitted Drude parameters of the 5×10^{-6} Torr $SrNbO_3$ film.

$\tilde{n}(\omega)$ Component	Free carrier density (cm^{-3})	Mobility (cm^2 V^{-1} s^{-1})
Ordinary (in-plane)	7.1×10^{21}	4.2
Extraordinary (out-of-plane)	6.8×10^{21}	8.1

Supplementary Note 1: The photocatalytic hydrogen evolution performance of Pt loaded $SrNbO_3$

The most interesting potential application of $SrNbO_3$ is its usage as a photocatalyst to convert the solar energy into the chemical energy by photocatalytic hydrogen evolution reactions. In our report, the measurement of a typical time course of H_2 evolution in 100 ml aqueous oxalic acid solution (0.025M) with 50 mg $SrNbO_3$ with Pt loading (1 wt. %) was performed under visible light irradiation (> 410 nm). Supplementary Figure 12 shows that the hydrogen production can reach a rate of 0.5135±0.705 $\mu mol \cdot h^{-1}$ for 50 mg catalyst and 100 ml solutions. If we use the unit of $\mu mol \cdot g^{-1} \cdot h^{-1}$, the hydrogen production rate of Pt loaded $SrNbO_3$ is 10.27±1.41 $\mu mol \cdot g^{-1} \cdot h^{-1}$, which is smaller than that of the reported value (44.8±5.9 $\mu mol \cdot g^{-1} \cdot h^{-1}$)[6]. This can be attributed to the much smaller surface area of our $SrNbO_3$ powders without ball milling compared with that of the reported one after ball milling as the surface area could be increased by at least 30 times after ball milling. Another possible reason is that the volume of the solution (50 mg catalyst in100 ml solution) used in our measurement are much less than those in the reported one (200 mg catalyst and 200 ml solution) though the dependence is not linear. The hydrogen production without any irradiation is much smaller (2.16 $\mu mol \cdot g^{-1} \cdot h^{-1}$) than that under visible light irradiation though this number is unusually large compared to other catalysts and we are investigating this more in depth. This has been discussed in the main text.

Supplementary Note 2: Spectroscopic Ellipsometry Methods and Analysis

Spectroscopic ellipsometry (SE) measurements of the 5×10^{-6} Torr sample were performed at room temperature using Woollam V-VASE ellipsometer with an energy range of 0.6-6.5 eV. The measurements were taken at incident angles of 50°, 60°, and 70°. from the sample normal and a beam spot size of ~1-3 mm. The measurements resulted in the ellipsometric parameters of the sample $tan\Psi \ \exp(i\Delta) \equiv r_p/r_s$, where r_p and r_s are the p- and s-polarized component of the amplitude reflection coefficients, respectively[1].

The resulting ψ and Δ spectra of the 5×10^{-6} Torr sample were analyzed using Woollam WVASE32 and Woollam CompleteEase softwares to extract the complex dielectric function spectra, $\varepsilon(\omega) = \varepsilon_1(\omega) + i\varepsilon_2(\omega)$, of the film, where ω is the photon angular frequency. Since the 5×10^{-6} Torr sample is a thin film deposited on an $LaAlO_3$ substrate, it was modelled as a two-layer system[1]. The analysis was performed using two modes: (1) isotropic mode, where the $\varepsilon(\omega)$ along all directions is assumed to be the same, and (2) uniaxial anisotropic mode, where the $\varepsilon(\omega)$ along the out-of-plane (extraordinary) direction is assumed to be different from the $\varepsilon(\omega)$ along the in-plane (ordinary) directions. The fitting was performed until a least-chi-square fit was achieved. The fit showed that the thickness of the film was ~222 nm, with a thickness non-uniformity of ~13%.

From the analysis, it was found that the ψ and Δ spectra of the 5×10^{-6} Torr sample cannot be fitted properly using isotropic mode and can only be fitted well at all incident angles using uniaxial anisotropic mode (Supplementary Figure 6). This indicated that the 5×10^{-6} Torr film had a slight uniaxial anisotropy along the out-of-plane direction. The resulting ordinary (along in-plane directions) and extraordinary (along out-of-plane direction) $\varepsilon(\omega)$spectra of

the film are shown in Supplementary Figure 7. It can be seen that the ordinary (and extraordinary) $\varepsilon_1(\omega)$ of the film crosses zero at ~1.8 eV, further confirming that a plasmon resonance should exist near this energy value.

From $\varepsilon(\omega)$, the frequency-dependent complex refractive index of the film, $\tilde{n}(\omega) = n(\omega) + \kappa(\omega)$, was obtained using

$$n(\omega) = \sqrt{\frac{1}{2}[\sqrt{\varepsilon_1^2(\omega) + \varepsilon_2^2(\omega)} + \varepsilon_1(\omega)]} \quad (1)$$

and

$$\kappa(\omega) = \sqrt{\frac{1}{2}[\sqrt{\varepsilon_1^2(\omega) + \varepsilon_2^2(\omega)} - \varepsilon_1(\omega)]}. \quad (2)$$

The ordinary $n(\omega)$ and $\kappa(\omega)$ spectra of the film are shown in Fig.2 (c), while its extraordinary $n(\omega)$ and $\kappa(\omega)$ spectra are shown in Supplementary Figure 8 (a). Meanwhile, the loss function (LF) spectra of the film was obtained from $\varepsilon(\omega)$ using

$$LF = -Im[\varepsilon^{-1}(\omega)] = \frac{\varepsilon_2(\omega)}{\varepsilon_1^2(\omega)+\varepsilon_2^2(\omega)}, \quad (3)$$

and the ordinary and extraordinary LF spectra of the film are shown Fig.2 (d) and Supplementary Figure 8 (b), respectively. From the figures, it can be seen that the extraordinary $\tilde{n}(\omega)$ and LF spectra of the film are generally similar in shapes and photon energies as compared to their ordinary $\tilde{n}(\omega)$ and LF spectra, with differences only in relative peak heights. Because of this, only the ordinary $\tilde{n}(\omega)$ and LF spectra of the film are shown in Fig.2 (d) for clarity. From $\tilde{n}(\omega)$, the normal-incident reflectivity, $R(\omega)$, of the film (Fig.2 (e)) was extracted using Fresnel equations, while the Kubelka-Munk function[2, 3],

$f_{KM}(\omega)$, of the film (Fig.2 (e)) was obtained using

$$f_{KM}(\omega) = \frac{[1-R(\omega)]^2}{2R(\omega)} \tag{S4}$$

We present the resulting parameters from the Drude fit of both the ordinary and extraordinary components of the complex refractive index, $\tilde{n}(\omega) = n(\omega) + \kappa(\omega)$, of the 5×10^{-6} Torr $SrNbO_3$ film in Supplementary Table 3 below. Note that the fitted free carrier density and mobility are within the same order as the values measured using transport measurements. The background dielectric constant used for the fitting is the vacuum permittivity, ε_0, while the actual energy-dependent complex dielectric function of the film can be seen in Supplementary Figure 7. As we mentioned in the Methods section, in the fitting the sample is geometrically modelled as a two-layered system: $SrNbO_3$ film on $LaAlO_3$ substrate.

Supplementary Note 3: Interpretation of differential reflection (DR) peaks:

As we can see in Fig. 4 (a), there is a DR peak near 600 nm. However, we do not find such a peak near 600 nm (2.07 eV) in the absorbance spectrum (Fig. 2(b)).

There are three possible mechanisms to explain this but only one fits our data.

<u>Model 1, Interband transitions above the Fermi level:</u> After pump pulse excitation, the excited electrons might be in some fully unoccupied band (we will call this B_1) above the CB band. If so, there should be a further higher fully unoccupied band (B_2) which is about 2.07 eV above the B_1 band in order to account for our data. However, in the band structure of $SrNbO_3$ in Fig. 4(b), there is no such B_2 band above the B_1 band. However, even if B_2 band exists, the hot electron relaxation from B_2 to lower energy levels would occur on a timescale of picoseconds[4] which is not consistent with our experimental results where a strong signal near 600 nm at a 1000.0 ps delay is seen (Supplementary Figure 10(a)) (The intensity of the DR signal at 1000.0 ps is similar to the intensity at 5.0 ps. This is because of an artifact spike around 25 ps in the dynamic curve. (This spike is due to a laser pulse reflected by the back side of a beam splitter in the pump beam optical path. However, this artifact does not affect the dynamics beyond 25ps where we have fitted all the curves). Thus, the band excitations above the Fermi level cannot account for our data.

<u>Model 2, Interband transitions below the Fermi level:</u> We need to assume that there may be some deep trapped states which are about 2.07 eV (~600 nm) above the B_{-1} band. The density of states of these deep trapped states should be dilute as we do not see any obvious features of in both the reflection and transmittance UV-Visible spectra (Fig. 2(b)). Therefore,

the gap between these deep trapped states and Fermi level must be ~ 1.85 eV (~670 nm), which can account for the negative peak in the DR spectrum in Fig. 4(a). When electrons in the deep trapped states are excited to the conductions band by the pump pulse, holes would be left in these states. When the probe pulse comes, the electrons in the B_{-1} band would be excited to the deep trapped states by the probe photons which will induce a positive peak (~600 nm) in the DR spectra. However, if the wavelength of pump light is larger than 670 nm (1.85 eV), such as 750 nm (1.65 eV) or 800 nm (1.55 eV), the pump photon will not sufficiently deplete the electrons in the deep trapped states. The amount of holes in the deep trapped states would then be too small to allow excited electrons from B_{-1} to the deep trapped states when the probe pulse arrives. Hence the positive peak near 600 nm should diminish and disappear. This prediction is not consistent with the experimental result shown in Fig. 4(a) in which the positive peak near 600 nm still exists independent of the pump wavelength. Hence the inter band transitions below the Fermi level cannot explain our data.

Model 3, High temperature electron induced thermal broadening of plasmons: This is the correct model and is described in the text. One may argue that usually the “bleaching” intensity of plasmon band should be larger than “darkening” intensity of the derivative peak, but here the intensity of the peak near 670 nm is smaller than that at 600 nm, as shown in Fig. 4(a). To solve this confusion, the differential transmission (DT, $\Delta T/T$) spectra were measured as shown in Supplementary Figure 10(b) with the pump light at 600 nm and various time delays. The 600 nm peak shifts to 560 nm in the DT spectrum which would not occur if this peak is due to interband electron transition process. In this spectrum, the peak intensity near 670 nm is about three times that of the derivative peak and the dynamics of

these two processes are similar to each other. The relatively low intensity of 670 nm peak in DR spectrum is a result of the reflection measurement method as the reflectivity goes to zero at 600nm as shown in Fig. 2(b). Therefore, the DT spectrum further proves that the 670 nm peaks in DR spectra can be attributed to the “bleaching” of the plasmon band while the 600 nm peaks are the derivative peaks due to the broadening of plasmon band (The reason why the DT spectra were not used in the manuscript is that the $LaAlO_3$ substrate can generate some negative signal near 600 and 760 nm in the DT spectrum and this could affect the measurement result and its analysis[5]. On the other hand, there is no contribution from $LaAlO_3$ in its DR spectrum).

This interpretation can also help one understand why the DR spectra with pump wavelength less than 670 nm are similar to those larger than 670 nm because the electrons can be heated independent of the pump wavelength.

Supplementary References

1. Fujiwara, H. *Spectroscopic Ellipsometry: Principles and Applications*. Wiley, 2007.
2. Born, M. & Wolf, E. *Principles of Optics: Electromagnetic Theory of Propagation, Interference and Diffraction of Light*. Cambridge University Press, 1997.
3. Kubelka, P. & Munk, F. An article on optics of paint layers. *Z Tech Phys* **12**(593-601), (1931).
4. Xing, G. *et al.* Long-range balanced electron- and hole-transport lengths in organic-inorganic CH3NH3PbI3. *Science* **342**(6156), 344-347 (2013).
5. Chen, J. Q. *et al.* Defect dynamics and spectral observation of twinning in single crystalline LaAlO3 under subbandgap excitation. *Appl. Phys. Lett.* **98**, 2-4 (2011).
6. Xu, X. Randorn, C. Efstathiou, P. & Irvine, J. T. S. A red metallic oxide photocatalyst. *Nat. Mater.* **11**, 595-598 (2012).
